# Intravascular Large B-cell Lymphoma in a Young Southeast Asian Male With Recurrent Strokes and Pulmonary Ground Glass Opacities With a Normal Chest Radiograph

**DOI:** 10.7759/cureus.66112

**Published:** 2024-08-04

**Authors:** Jiaxuan Liu, Chee Leong Cheng, Mariko Koh

**Affiliations:** 1 Respiratory Medicine, Sengkang General Hospital, Singapore, SGP; 2 Pathology, Singapore General Hospital, Singapore, SGP; 3 Respiratory and Critical Care Medicine, Singapore General Hospital, Singapore, SGP

**Keywords:** southeast asian, transbronchial lung biopsy, multiple pulmonary nodules, stroke, intravascular large b-cell lymphoma

## Abstract

Intravascular large B-cell lymphoma (IVLBCL) is a rare form of extranodal large B-cell lymphoma characterized by the growth of lymphoma cells within lumina of blood vessels, especially capillaries, which aggregate to form clots, resulting in organ ischemia. In Caucasians, it predominantly involves the central nervous system (CNS) and the skin, with the cutaneous variant carrying a better prognosis. Whereas in Asians it preferentially involves the bone marrow, liver, and spleen and is associated with hemophagocytic syndrome. We report a case of a young Asian male with neurological, pulmonary, and hepatosplenic involvement. He presented with recurrent strokes, chronic cough, and unintentional weight loss. The chest radiograph (CXR) on admission was clear. Magnetic resonance imaging (MRI) of the brain showed acute multifocal infarcts, and a whole-body computed tomography (CT) scan revealed upper-lobe predominant pulmonary ground glass opacities (GGOs) with mediastinal lymphadenopathy. Interestingly, a CXR performed one week after the CT scan remained clear. The positron emission tomography-computed tomography (PET-CT) showed hepatosplenic and adrenal involvement. The diagnosis was confirmed via a bronchoscopic approach. The patient received chemotherapy consisting of MR-CHOP (methotrexate, rituximab, cyclophosphamide, adriamycin, vincristine, and prednisolone), high-dose methotrexate, and intrathecal cytarabine, which led to complete remission. Subsequently, he underwent an autologous peripheral blood stem cell transplant. At the time of writing this case report, the patient is still in complete remission for three years after the initial diagnosis. As IVLBCL has a non-specific clinicoradiological presentation, it is important to suspect IVLBCL in patients with an atypical neurological and pulmonary presentation in the presence of raised serum lactate dehydrogenase (LDH) and to consider a CT scan of the thorax if CXR is normal.

## Introduction

Intravascular large B-cell lymphoma (IVLBCL) is a rare form of extra-nodal large B-cell lymphoma with an estimated annual incidence of about 0.5-1 in 1,000,000 people [1]. It is characterized by the growth of lymphoma cells within the lumina of blood vessels, especially capillaries, manifesting as disseminated ischemic lesions [2]. In Caucasians, it predominantly involves the central nervous system (CNS) and the skin, with the cutaneous variant carrying a better prognosis. Whereas in Asians it preferentially involves the bone marrow, liver, and spleen and is associated with hemophagocytic syndrome. As IVLBCL has a non-specific clinicoradiological presentation and carries a poor prognosis due to its aggressive nature and rarity, we report this case to raise its awareness among physicians. We describe a Southeast Asian patient presenting with recurrent strokes, chronic cough, and weight loss. His clinical presentation was unique. Although his computed tomography (CT) scan showed multiple ground glass opacities, his chest radiographs (CXRs) remained clear. The diagnosis was promptly achieved via endobronchial ultrasound-guided biopsy of mediastinal lymph nodes and transbronchial lung biopsy with subsequent successful treatment.

## Case presentation

A 41-year-old Cambodian male presented to the emergency department with the sudden onset of right-sided limb weakness and headache. He reported four months of dry cough and unintentional weight loss of more than 20 kilograms within a year.

The patient had well-controlled hypertension. He had no family history of malignancy or autoimmune diseases. His father had been treated for pulmonary tuberculosis. He was married and worked for a non-governmental organization in Singapore. He did not smoke, take recreational drugs, or participate in promiscuous activities. He was afebrile, and his vital signs were normal. On examination, he had mild right upper and lower limb weakness; otherwise, the sensation was intact. Cerebellar and cranial nerve examinations were normal. His lungs were clear, and there were no palpable cervical lymph nodes. The rest of the systemic examination was unremarkable.

**Figure 1:**
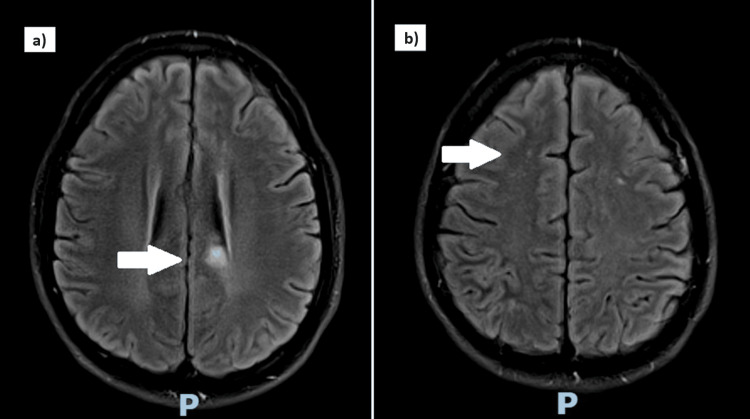
Diffusion-weighted images of the brain MRI show acute multifocal infarcts in a) the left aspect of the splenium of the corpus callosum (arrow) and b) the right parietal subcortical white matter with no intra- or extra-cranial stenosis (arrow).

A CT scan of the brain showed a right parietal hyperdensity of indeterminate etiology. A subsequent brain magnetic resonance imaging (MRI) and magnetic resonance angiography (MRA) revealed acute multifocal infarcts in the left splenium of the corpus callosum and right parietal subcortical white matter with no intra- or extra-cranial stenosis (Figure 1). The laboratory results on admission are shown in Table 1. An extensive young stroke workup was normal. This included immunological studies (anti-nuclear antibody, anti-double stranded DNA antibody, anti-neutrophil cytoplasmic antibodies), thrombophilia screen (anti-cardiolipin and lupus anticoagulant antibodies, anti-thrombin antibody, proteins C and S), serum flow cytometry, microbiological studies (human immunodeficiency virus, hepatitis B and C), serum homocysteine level, urine and serum drug screen, transthoracic echocardiography with bubble contrast, and 24-hour holter. Chest radiographs and electrocardiograms on admission were normal.

**Table 1:** Basic laboratory tests

Laboratory test	Result	Reference range
White cell count (X 10^9 ^/L)	10.2	4 – 10
Haemoglobin (g/dL)	13.2	12-16
Platelet (X 10^9 ^/L)	197	140- 440
Absolute eosinophil count (X 10^9 ^/L)	11.2	0.04 – 0.44
No circulating lymphoma cells seen in the peripheral blood		
Creatinine (µmol/L)	152	45 - 84
Lactate dehydrogenase (U/L)	1705	135 - 214
Partial thromboplastin time (PTT, sec)	29	25.7 – 32.9
Prothrombin time (PT, sec)	11.1	9.9 – 11.4
Aspartate transaminase (U/L)	27	<= 35
Alanine transaminase (U/L)	14	<= 35
C-reactive protein (mg/L)	6	<= 4.9
Triglycerides (mmol/L)	1.8	Desirable level: 1.7 – 2.2
Cholesterol, lactate dehydrogenase (LDH, mmol/L)	2.4	Desirable level: 2.6 – 3.3

**Figure 2:**
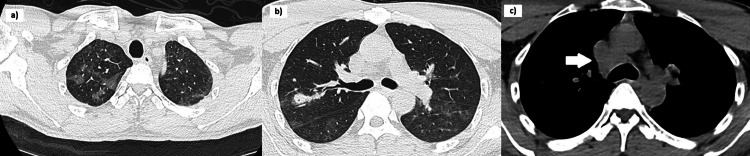
A CT scan of the thorax, abdomen, and pelvis shows ground glass opacities mainly distributed in a) both upper lobes, b) the superior segment of the lower lobes, and c) enlarged right lower paratracheal lymph nodes of 1.6 cm.

**Figure 3:**
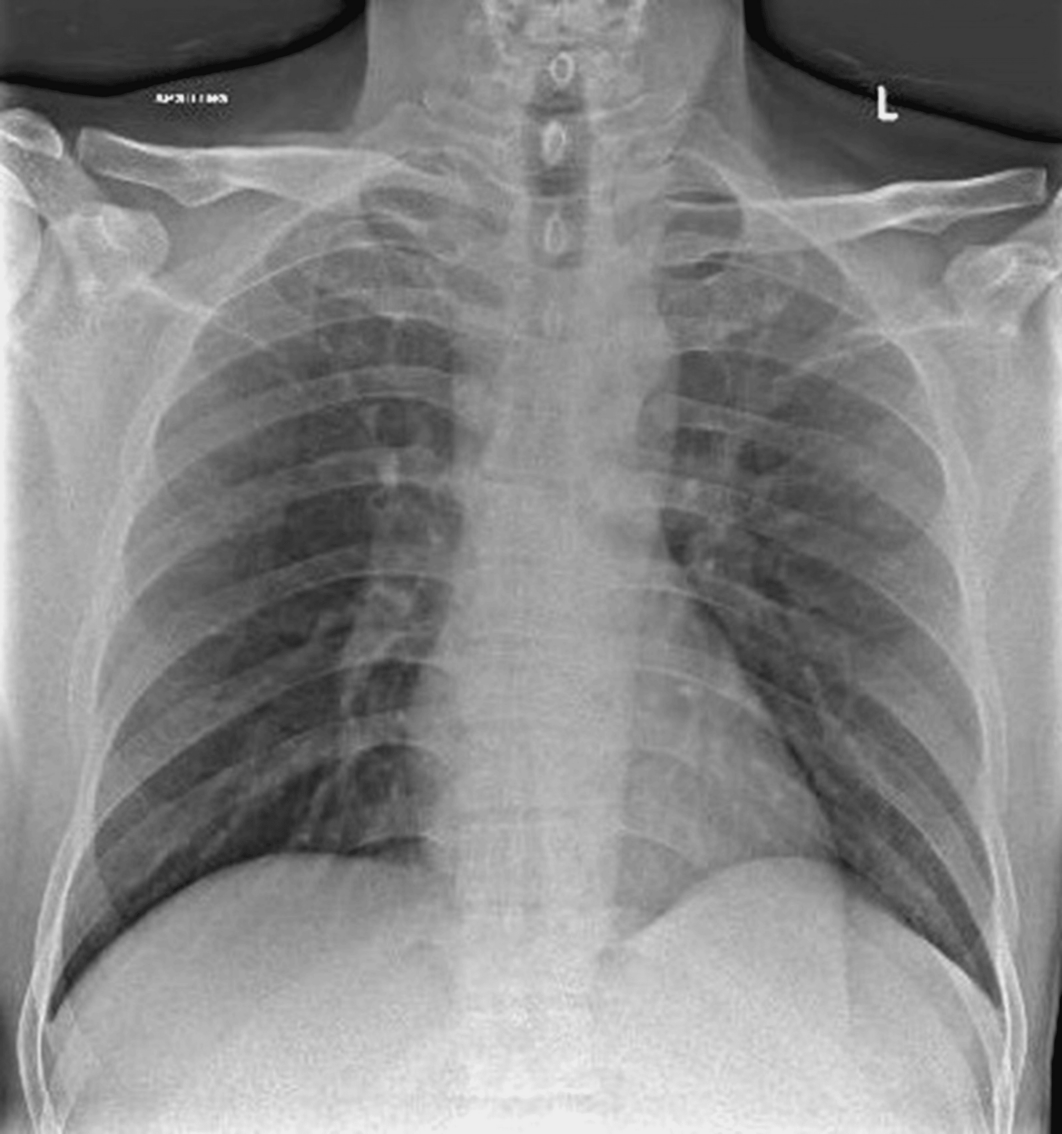
Chest radiograph obtained one week after CT scan of the thorax was performed

Five days later, the patient developed a worsening headache. Repeated brain MRI and MRA showed new multi-territorial infarcts. Cerebral spinal fluid analysis revealed a normal protein level, cell count, and microbiological studies; there were no malignant cells, and flow cytometry was negative. A CT scan of the thorax, abdomen, and pelvis performed three days later revealed bilateral peri-bronchovascular ground glass opacities (GGOs) seen predominantly in the upper lobes and superior segment of the lower lobes and enlarged right lower paratracheal lymph nodes (Figure 2). There was no hepatosplenomegaly or adrenal involvement. A CXR performed one week later for a fever spike remained clear (Figure 3).

**Figure 4:**
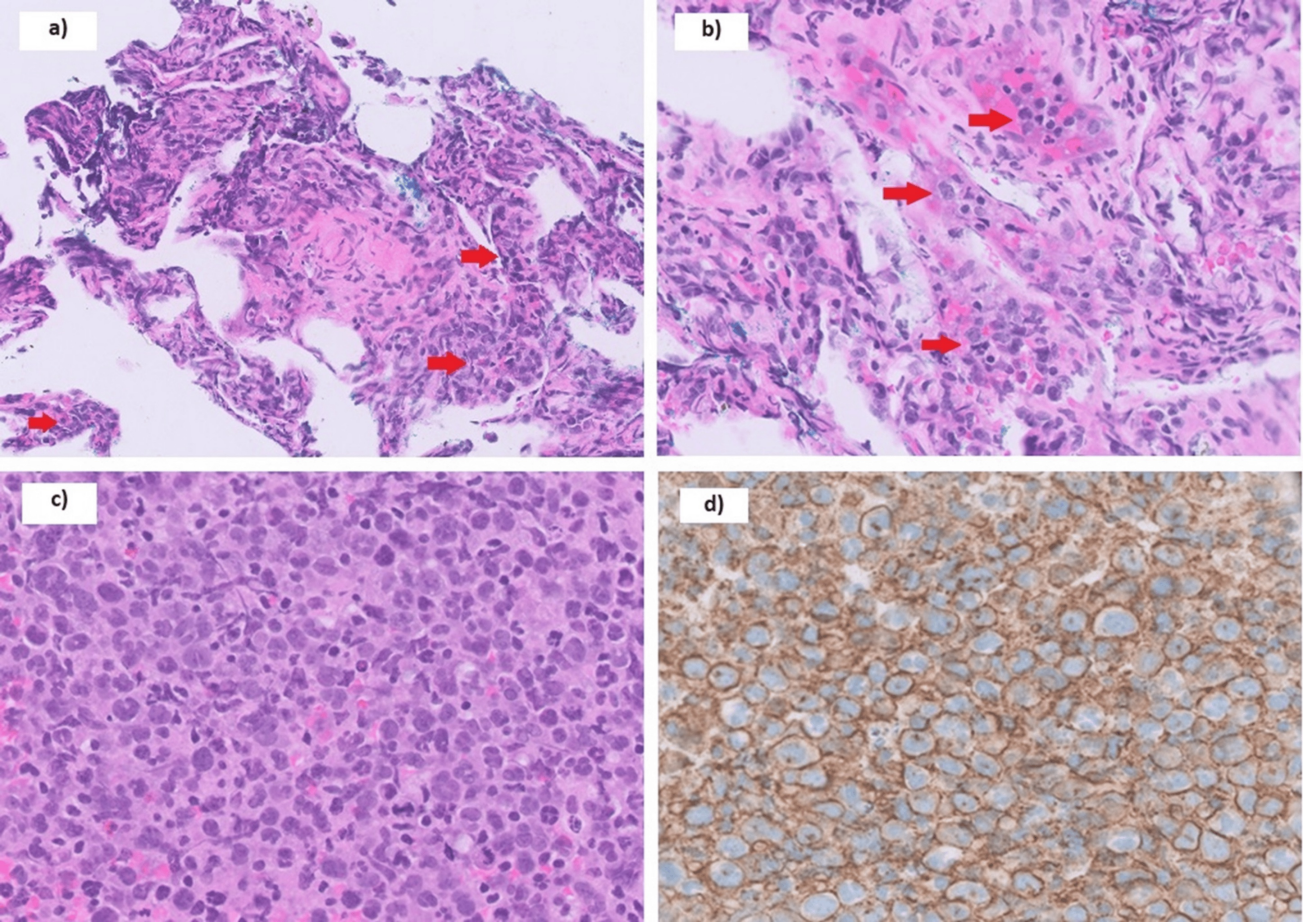
a) Lung biopsy shows intravascular infiltrate of atypical medium to large cells (H&E, 400x magnification); b) Lung biopsy shows intravascular infiltrate of atypical medium to large cells (arrow) (H&E, 600x magnification); c) Lymph node biopsy shows diffuse infiltrate of large lymphoid cells with variably irregular nuclei. Mitoses are readily seen (H&E, 600x magnification); d) Lymph node biopsy shows the diffuse infiltrate of large lymphoid cells to be CD20-positive large B-cells, consistent with diffuse large B-cell lymphoma. Cytogenetics studies by fluorescence in situ hybridization (FISH) only show BCL6 rearrangements without MYC or BCL2 rearrangements (CD20, 600x magnification)

**Figure 5:**
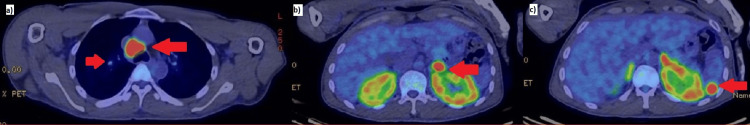
The PET-CT shows fluorodeoxyglucose (FDG) avidity in a) right upper lobe ground glass opacities and station 4R lymph node, b) left adrenal gland, and c) left spleen.

The patient underwent bronchoscopy with endobronchial ultrasound-guided biopsy of the right paratracheal lymph nodes, transbronchial lung biopsy, and bronchoalveolar lavage of the right upper lobe GGOs. Histology of lung biopsy revealed increased cellularity in the alveolar septa due to intravascular infiltrates of atypical medium to large cells (H&E, 400x magnification) (Figure 4a). Intravascular infiltrates of atypical medium-to-large cells were found to be CD20-positive lymphomatous large B-cells, occurring in rows and elongated aggregates (H&E, 600x magnification) (Figure 4b). Lymph node biopsy showed diffuse infiltrates of large lymphoid cells with variably irregular nuclei (H&E, 600x magnification) (Figure 4c) and was CD20 positive (CD20, 600x magnification) (Figure 4d). A cytogenetics study by fluorescence in situ hybridization (FISH) showed BCL6 rearrangements without MYC or BCL2 rearrangements. Bronchoalveolar lavage showed no malignant cells, and microbiological studies including *Mycobacterium tuberculosis* were unyielding. A diagnosis of IVLBCL was made. Subsequent positron emission tomography-computed tomography (PET-CT) scans showed fluorodeoxyglucose (FDG) avidity in the corresponding areas of pulmonary abnormalities noted on CT and in the adrenal glands, spleen, and bone marrow (Figure 5). However, bone marrow trephine and aspirate showed no lymphomatous involvement.

**Figure 6:**
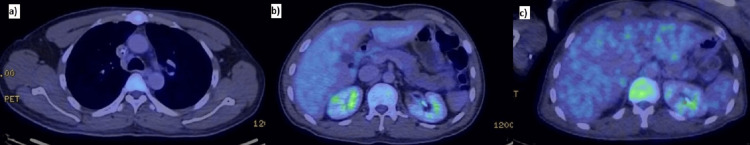
After the third cycle of MR-CHOP (methotrexate, rituximab, cyclophosphamide, adriamycin, vincristine, and prednisolone) chemotherapy, repeat PET-CT showed resolution of the previously seen fluorodeoxyglucose (FDG) avidities in the a) right upper lobe ground glass opacities and station 4R lymph node, b) left adrenal gland, and c) left spleen. R: right

The patient completed six cycles of MR-CHOP (methotrexate, rituximab, cyclophosphamide, adriamycin, vincristine, and prednisolone) with a dose of intrathecal cytarabine during the first cycle. Serum lactate dehydrogenase (LDH) normalized by the end of the second cycle. A PET-CT after the third cycle showed complete resolution of the FDG-avid pulmonary GGOs and adrenal and splenic involvement (Figure 6). The patient subsequently underwent autologous hematopoietic stem cell transplant. At this time of writing, the patient is still in complete remission for three years after the initial diagnosis.

## Discussion

Intravascular large B-cell lymphoma manifests in two variants: the 'Western' type primarily affects the central nervous system and skin, while the 'Asian' variant mainly targets the liver, spleen, and bone marrow, often linked with hemophagocytic syndrome [2]. Despite our patient being Asian, his presentation was unusual, involving neurological and pulmonary symptoms. From a literature review, symptoms are non-specific and vary depending on organ involvement.

Interestingly, his CXRs were clear despite the presence of pulmonary GGOs on the CT scan of the thorax. Most case reports and case series of IVLBCL with pulmonary involvement did not mention any CXR findings. However, we found a case series published by Nguyen et al., where three patients had dyspnea, B symptoms, and abnormal CT thorax findings that included intrathoracic lymphadenopathy, GGOs, and mosaic attenuation, but CXRs were normal [3]. Two were diagnosed via surgical lung biopsy and one during autopsy. We opine that obtaining an early CT scan of the thorax is important if the patient has respiratory and B symptoms and normal CXR, as CXR might miss CT findings and delay diagnosis. Another significant finding is that our patient and some case reports had predominantly upper lobe pathologies, which is helpful in narrowing down differentials [4-6]. A possible explanation is that the lymphoma cells have the highest tendency to clot in lung apices, where the blood flow rate and lymphatic clearance are lowest, as both are driven by perfusion and respiratory excursion [7]. Other differentials of upper lobe predominant GGOs include pulmonary metastases and pulmonary tuberculosis, which is prevalent in Southeast Asia. Given his history of chronic cough, weight loss, and prior tuberculosis exposure, pulmonary tuberculosis is a significant consideration.

We opine that PET-CT is helpful in guiding diagnostic modality, especially for cases with neurological manifestations where safer alternatives to brain biopsy are preferred. Raised serum LDH is not specific for IVLBCL, but it raises suspicion.

The pulmonary GGOs correlated histopathologically with the neoplastic lymphocytes filling the blood vessels to cause alveolar septal widening [8]. These neoplastic lymphocytes sequester within vessel lumens due to a lack of cell surface proteins, CD29 and CD54, which are required for lymphocyte transvascular migration [9].

## Conclusions

Diagnosing IVLBCL can be challenging due to its rarity and aggressive nature. When evaluating a patient presenting with young-onset stroke alongside constitutional symptoms and elevated serum LDH, it is crucial to consider IVLBCL among other potential diagnoses. In cases where a chest X-ray is normal, performing a CT scan of the thorax is advisable as it can reveal findings that might be missed on the X-ray, thus preventing diagnostic delays. Notably, our case and several reported cases have shown a similar pattern of upper lobe predominant GGOs on CT scans of the thorax, which can aid in narrowing down the diagnosis. In our case, it remains important to rule out pulmonary tuberculosis, particularly in Southeast Asia. While core biopsy is usually the preferred method for suspected lymphoma, bronchoscopy with transbronchial lung biopsy and endobronchial ultrasound-guided fine needle aspiration of lymph nodes can be considered if other safer biopsy sites are not available, as demonstrated in our case report.
